# Publisher Correction: Functional synapses between neurons and small cell lung cancer

**DOI:** 10.1038/s41586-025-09638-z

**Published:** 2025-09-19

**Authors:** Vignesh Sakthivelu, Anna Schmitt, Franka Odenthal, Kristiano Ndoci, Marian Touet, Ali H. Shaib, Abdulla Chihab, Gulzar A. Wani, Pascal Nieper, Griffin G. Hartmann, Isabel Pintelon, Ilmars Kisis, Maike Boecker, Naja M. Eckert, Manoela Iannicelli Caiaffa, Olta Ibruli, Julia Weber, Roman Maresch, Christina M. Bebber, Ali Chitsaz, Anna Lütz, Mira Kim Alves Carpinteiro, Kaylee M. Morris, Camilla A. Franchino, Jonas Benz, Laura Pérez-Revuelta, Jorge A. Soriano-Campos, Maxim A. Huetzen, Jonas Goergens, Milica Jevtic, Hannah M. Jahn-Kelleter, Hans Zempel, Aleksandra Placzek, Alexandru A. Hennrich, Karl-Klaus Conzelmann, Hannah L. Tumbrink, Pascal Hunold, Joerg Isensee, Lisa Werr, Felix Gaedke, Astrid Schauss, Marielle Minère, Marie Müller, Henning Fenselau, Yin Liu, Alena Heimsoeth, Gülce S. Gülcüler Balta, Henning Walczak, Christian Frezza, Ron D. Jachimowicz, Julie George, Marcel Schmiel, Johannes Brägelmann, Tim Hucho, Silvia von Karstedt, Martin Peifer, Alessandro Annibaldi, Robert Hänsel-Hertsch, Thorsten Persigehl, Holger Grüll, Martin L. Sos, Guido Reifenberger, Matthias Fischer, Dirk Adriaensen, Reinhard Büttner, Julien Sage, Inge Brouns, Roland Rad, Roman K. Thomas, Max Anstötz, Silvio O. Rizzoli, Matteo Bergami, Elisa Motori, Hans Christian Reinhardt, Filippo Beleggia

**Affiliations:** 1 Department of Translational Genomics, Faculty of Medicine and University Hospital Cologne, University of Cologne, Cologne, Germany; 2 Department I of Internal Medicine, Faculty of Medicine and University Hospital Cologne, University of Cologne, Cologne, Germany; 3 Mildred Scheel School of Oncology Aachen Bonn Cologne Düsseldorf (MSSO ABCD), Faculty of Medicine and University Hospital Cologne, University of Cologne, Cologne, Germany; 4 Institute of Virology, Faculty of Medicine and University Hospital Cologne, University of Cologne, Cologne, Germany; 5 Institute of Biochemistry, University of Cologne, Cologne, Germany; 6 Cologne Excellence Cluster on Cellular Stress Response in Aging-Associated Diseases (CECAD), University of Cologne, Cologne, Germany; 7 Department of Hematology and Stem Cell Transplantation, University Hospital Essen, Essen, Germany; 8 Department of Neuro- and Sensory Physiology, University Medical Center Göttingen, Göttingen, Germany; 9 Department of Pediatrics, Stanford University, Stanford, CA USA; 10 Department of Genetics, Stanford University, Stanford, CA USA; 11 Laboratory of Cell Biology and Histology, Department of Veterinary Sciences, University of Antwerp, Antwerp, Belgium; 12 Antwerp Centre for Advanced Microscopy (ACAM), University of Antwerp, Antwerp, Belgium; 13 Institute of Molecular Oncology and Functional Genomics, School of Medicine, Technical University of Munich, Munich, Germany; 14 Max Planck Institute for Biology of Ageing, Cologne, Germany; 15 Center for Molecular Medicine Cologne (CMMC), Faculty of Medicine and University Hospital Cologne, University of Cologne, Cologne, Germany; 16 Institute of Human Genetics, Faculty of Medicine and University Hospital Cologne, University of Cologne, Cologne, Germany; 17 Virology, Max von Pettenkofer Institute, Faculty of Medicine and Gene Center, Ludwig Maximilians University, Munich, Germany; 18 Molecular Pathology, Institute of Pathology, Faculty of Medicine and University Hospital Cologne, University of Cologne, Cologne, Germany; 19 Translational Pain Research, Department of Anesthesiology and Intensive Care Medicine, Faculty of Medicine and University Hospital Cologne, University of Cologne, Cologne, Germany; 20 Pain Center, Department of Anesthesiology and Intensive Care Medicine, Faculty of Medicine and University Hospital Cologne, University of Cologne, Cologne, Germany; 21 Department of Experimental Pediatric Oncology, University Children’s Hospital of Cologne, Medical Faculty, University of Cologne, Cologne, Germany; 22 Research Group Synaptic Transmission in Energy Homeostasis, Max Planck Institute for Metabolism Research, Cologne, Germany; 23 Policlinic for Endocrinology, Diabetes and Preventive Medicine, University Hospital Cologne, Cologne, Germany; 24 Janelia Research Campus, Howard Hughes Medical Institute, Ashburn, VA USA; 25 Cell Death, Inflammation and Immunity Laboratory, Institute of Biochemistry I, Center for Biochemistry, Faculty of Medicine, University of Cologne, Cologne, Germany; 26 Centre for Cell Death, Cancer and Inflammation, UCL Cancer Institute, University College London, London, UK; 27 Institute for Metabolomics in Ageing, University of Cologne, Faculty of Medicine and University Hospital Cologne, Cologne Excellence Cluster on Cellular Stress Responses in Aging-Associated Diseases (CECAD), Cologne, Germany; 28 Institute of Genetics, University of Cologne, Faculty of Mathematics and Natural Sciences, Cologne Excellence Cluster on Cellular Stress Responses in Aging-Associated Diseases (CECAD), Cologne, Germany; 29 Department of Otorhinolaryngology, Head and Neck Surgery, University Hospital Cologne, Cologne, Germany; 30 Institute of Pathology, Faculty of Medicine and University Hospital Cologne, University of Cologne, Cologne, Germany; 31 Institute for Diagnostic and Interventional Radiology, Faculty of Medicine and University Hospital Cologne, University of Cologne, Cologne, Germany; 32 Department of Translational Oncology, German Cancer Consortium (DKTK), partner site Munich, a partnership between DKFZ and Ludwig-Maximilians-Universität München (LMU), Munich, Germany; 33 Department of Medicine III, LMU University Hospital Munich, Munich, Germany; 34 Institute of Neuropathology, Medical Faculty, Heinrich Heine University and University Hospital Düsseldorf, Düsseldorf, Germany; 35 Institute of Anatomy II, Medical Faculty, University Hospital Düsseldorf, Heinrich Heine University, Düsseldorf, Germany; 36 Center for Biostructural Imaging of Neurodegeneration, University Medical Center Göttingen, Göttingen, Germany; 37 Cluster of Excellence ‘Multiscale Bioimaging: from Molecular Machines to Networks of Excitable Cells’ (MBExC), University of Göttingen, Göttingen, Germany; 38 Institute of Genetics, University of Cologne, Cologne, Germany; 39 Faculty of Medicine and University Hospital Cologne, University of Cologne, Cologne, Germany; 40 West German Cancer Center, University Hospital Essen, Essen, Germany; 41 DKTK, partner site Essen, University Hospital Essen, Essen, Germany; 42 Center for Molecular Biotechnology, University Hospital Essen, Essen, Germany

**Keywords:** Cancer microenvironment, Cancer genomics, Neuroscience, Small-cell lung cancer, Targeted therapies

Correction to: *Nature* 10.1038/s41586-025-09434-9 Published online 10 September 2025

In the version of the article initially published, there was an error in Manoela Iannicelli Caiaffa’s name (“Iannicelli” appeared as “Ianicelli”). This has now been amended in the HTML and PDF versions of the article.

