# Supplementary material for: Functional synapses between neurons and small cell lung cancer
Source: Nature. 2025 Sep 10;646(8087):1243–53. doi: 10.1038/s41586-025-09434-9 (PMC12571904; doi:10.1038/s41586-025-09434-9)
Supplement: Supplementary file 2 — Reporting Summary [file 41586_2025_9434_MOESM2_ESM.pdf]

nature portfolio

Corresponding author(s): Filippo Beleggia
Christian Reinhardt
Elisa Motori
Matteo Bergami
Silvio Rizzoli
Max Anstötz

Last updated by author(s): Jun 17, 2025

Reporting Summary

Nature Portfolio wishes to improve the reproducibility of the work that we publish. This form provides structure for consistency and transparency in reporting. For further information on Nature Portfolio policies, see our Editorial Policies and the Editorial Policy Checklist.

Statistics

For all statistical analyses, confirm that the following items are present in the figure legend, table legend, main text, or Methods section.

n/a	Confirmed	
☐	☒	The exact sample size (*n*) for each experimental group/condition, given as a discrete number and unit of measurement
☐	☒	A statement on whether measurements were taken from distinct samples or whether the same sample was measured repeatedly
☐	☒	The statistical test(s) used AND whether they are one- or two-sided<br>*Only common tests should be described solely by name; describe more complex techniques in the Methods section.*
☒	☐	A description of all covariates tested
☐	☒	A description of any assumptions or corrections, such as tests of normality and adjustment for multiple comparisons
☐	☒	A full description of the statistical parameters including central tendency (e.g. means) or other basic estimates (e.g. regression coefficient) AND variation (e.g. standard deviation) or associated estimates of uncertainty (e.g. confidence intervals)
☐	☒	For null hypothesis testing, the test statistic (e.g. *F*, *t*, *r*) with confidence intervals, effect sizes, degrees of freedom and *P* value noted<br>*Give P values as exact values whenever suitable.*
☒	☐	For Bayesian analysis, information on the choice of priors and Markov chain Monte Carlo settings
☒	☐	For hierarchical and complex designs, identification of the appropriate level for tests and full reporting of outcomes
☐	☒	Estimates of effect sizes (e.g. Cohen's *d*, Pearson's *r*), indicating how they were calculated

Our web collection on statistics for biologists contains articles on many of the points above.

Software and code

Policy information about availability of computer code

Data collection: Electron micrographs were acquired with DigitalMicrograph (Gatan). Electrophysiology data were acquired using Signal (version 6.0, Cambridge Electronic, Cambridge, UK), Hokawo (version 2.8, Hamamatsu, Geldern, Germany), Igor Pro (version 32 7.01, WaveMetrics, Lake Oswego, OR, USA), Clampex (version 10.7.0.3, Molecular Devices, LLC). Imaging during electrophysiological recording was acquired with Micro-Manager (version 2.0.0, Open Source, UCSF). Electron micrographs were taken with DigitalMicrograph v3.32.2403.0 (Gatan). Tomograms were acquired using SerialEM v3.7.11

Data analysis	Genomic and expression data were processed using BWA version 0.7.15, samtools version 1.3.1, liftOver v385, GATK version 4.1.3.0, Annovar version 2018Apr16, STAR versions 2.4.2a, 2.5.3a and V_2.7.10b , HTSeq version 0.6.1p1, RNA-SeQC version 1.1.9. scRNAseq data was processed with the PARSE pipeline version 1.1.1. MRI images were analyzed with Horos version 3.0, with the package Export Rois version 2.0. Data was analyzed using Python version 3.8, 3.9 or 3.10 with the packages pandas version 1.1.4, numpy version 1.20.2, scipy version 1.6.3, statsmodels version 0.12.2, datashader version 0.12.1, matplotlib version 3.4.2, seaborn version 0.11.0, lifelines version 0.25.6, scanpy version 1.9.3, cellbender version 0.3.0, doubletdetection version 4.2. Analysis of electrophysiological data was performed with Clampfit (version 11.2.2.17, Molecular Devices, LLC). Pearson correlation analysis was performed with Matlab (The Mathworks, Inc., version 2023b). Tomograms were reconstructed with IMOD v4.11.7. The 3D reconstruction tomograms was performed with Imaris v10.2.0 (Oxford Instruments). Segmentation of EM structures was performed with Microscopy Image Browser (MIB, version 2.84). Registration of CLEM images was performed with the plugin EC-CLEM v1.1.0.0 from the software ICY v2.5.2.0. Image analysis was performed with ImageJ v1.54h, the Cell Counter plugin v3.0.0 and Fiji v2.14.0. Python scripts generated in this study are available from github (https://github.com/beleggia-lab/neuron-to-SCLC-synapses) and Zenodo (https://doi.org/10.5281/zenodo.15667860).

For manuscripts utilizing custom algorithms or software that are central to the research but not yet described in published literature, software must be made available to editors and reviewers. We strongly encourage code deposition in a community repository (e.g. GitHub). See the Nature Portfolio guidelines for submitting code & software for further information.

Data

Policy information about availability of data

All manuscripts must include a data availability statement. This statement should provide the following information, where applicable:

- Accession codes, unique identifiers, or web links for publicly available datasets
- A description of any restrictions on data availability
- For clinical datasets or third party data, please ensure that the statement adheres to our policy

Reference genomes were downloaded from GDC (TCGA GRCh38.d1.vd1, https://api.gdc.cancer.gov/data/254f697d-310d-4d7d-a27b-27fbf767a834), from Ensembl (https://www.ensembl.org, GRCm38.102 and GRCm39.110) and from GTEx (https://www.gtexportal.org, Homo_sapiens_assembly38_noALT_noHLA_noDecoy_ERCC.fasta). Gene annotations were downloaded from gencode (vM23 and v22, https://www.gencodegenes.org/). Orthology mapping was downloaded from the HGNC database (https://www.genenames.org/, downloaded January 6th 2020). Mutation data were downloaded from the supplementary tables of the referenced publications or from the CCLE website (Cell_lines_annotations_20181226.txt and CCLE_DepMap_18q3_maf_20180718.txt, https://portals.broadinstitute.org/ccle/). TCGA expression data were downloaded from the Genomic Data Commons Data Portal (v27, https://portal.gdc.cancer.gov). GTEx expression data were downloaded from the GTEx database (v8, https://gtexportal.org). Gene Ontology (GO) data were downloaded from the GO website (v2020-09-10, http://geneontology.org). ChIP-seq data were downloaded from the CISTROME database (v2, http://cistrome.org/db, accessed November 27th 2019). ScRNA-seq data, as well as the corresponding metadata were downloaded from Synapse (Synapse:syn21560406, https://www.synapse.org/) and CZ cellxgene (https://datasets.cellxgene.cziscience.com/7a30310a-2239-4d84-b99e-a12456c2fe19.h5ad). PhyloP conservation tracks across 470 mammalian genomes were downloaded from UCSC (hg38.470way.phyloP, https://genome.ucsc.edu/). Uniprot Knowledgebase annotations were downloaded from the Uniprot website (v2022_5, https://www.uniprot.org). Raw sequencing data from the piggyBac screen and murine snRNAseq are available through the Sequence Read Archive (SRA, https://www.ncbi.nlm.nih.gov/sra) under accessions PRJNA1275653 and PRJNA1276342, respectively. A scanpy data object of the snRNAseq dataset is available at Zenodo (https://doi.org/10.5281/zenodo.15647008). The full analyzed data from our whole-genome analyses are available in the supplementary information tables and the source data for all figures is provided in the online version of this manuscript.

Research involving human participants, their data, or biological material

Policy information about studies with human participants or human data. See also policy information about sex, gender (identity/presentation), and sexual orientation and race, ethnicity and racism.

Reporting on sex and gender	Human samples were not stratified by sex or gender
Reporting on race, ethnicity, or other socially relevant groupings	Human samples were not grouped based on race, ethnicity or other socially relevant grouping
Population characteristics	No covariate analysis was performed
Recruitment	Patients were recruited as part of the Biomasota study (13-091, 2016)
Ethics oversight	Patients consented to the use of their tissue specimens and approval was obtained by the Ethics Committee of the University of Cologne

Note that full information on the approval of the study protocol must also be provided in the manuscript.

Field-specific reporting

Please select the one below that is the best fit for your research. If you are not sure, read the appropriate sections before making your selection.

☒ Life sciences ☐ Behavioural & social sciences ☐ Ecological, evolutionary & environmental sciences

For a reference copy of the document with all sections, see nature.com/documents/nr-reporting-summary-flat.pdf

Life sciences study design

All studies must disclose on these points even when the disclosure is negative.

Sample size	Sample sizes for animal studies was selected based on power analysis. Sample sizes for other experiments were chosen based on previous experience with cancer cell lines and neuronal cultures (e.g. PMID: 30612738, 25661179), in order to capture the technical and biological variability of the different experimental settings. All the sample sizes are stated in the figure legends, main text or methods sections.
Data exclusions	No data were excluded from analysis.
Replication	All experimental findings were reproducible across at least two replicates.
Randomization	The allocation of samples to experimental groups was randomized. The neuronal batches could not each be tested with every cell line but the allocation of cell lines to neuronal batches was random.
Blinding	The evaluation of termination criteria for survival analysis of the mice was not blinded as the scientists also performed treatments which could not be blinded due to the different dosing schedules. All other data collection and data analysis was performed blindly or with automated pipelines.

Reporting for specific materials, systems and methods

We require information from authors about some types of materials, experimental systems and methods used in many studies. Here, indicate whether each material, system or method listed is relevant to your study. If you are not sure if a list item applies to your research, read the appropriate section before selecting a response.

Materials & experimental systems

n/a	Involved in the study
☐	☒ Antibodies
☐	☒ Eukaryotic cell lines
☒	☐ Palaeontology and archaeology
☐	☒ Animals and other organisms
☒	☐ Clinical data
☒	☐ Dual use research of concern
☒	☐ Plants

Methods

n/a	Involved in the study
☒	☐ ChIP-seq
☒	☐ Flow cytometry
☒	☐ MRI-based neuroimaging

Antibodies

Antibodies used

Chicken anti-GFP 1:500 (Alves Labs, Cat# GFP-1020)
Rabbit polyclonal anti-RFP 1:500 (Rockland, Cat#600401379)
Chicken anti-MAP2 1:500 (Abcam, Cat# ab5392)
Mouse monoclonal anti-vGluT1 1:500 (Synaptic Systems, Cat# 135 311)
Rabbit polyclonal anti-Homer1 1:500 (Synaptic Systems, Cat# 160 003)
Mouse monoclonal anti-Bassoon 1:500 (Synaptic Systems, Cat# 141 111)
goat anti-CGRP 1:1000 (Abcam, #ab36001)
rabbit GAP43 1:2000 (Novus Biologicals, #NB300-143)
chicken anti-GFP 1:500 (Abcam, #13970)
rabbit PGP9.5 1:2000 (Abcam, #ab108986)
rabbit anti-P2X3 1:1000 (Chemicon, #AB5895)
rat anti-SP 1:200 (Biogenesis, 8450-0505)
guinea-pig anti-SYP 1:4000 (Synaptic Systems, #101002)
rabbit anti-VGluT1 1:250 (Synaptic Systems, #135303)
mouse anti-Bassoon 1:500 (Enzo, ADI-VAM-PS003-F, cat# SAP7F407)
AlexaFluor 488 Donkey anti-Chicken 1:1000 (Jackson Immuno Research Labs, Cat# 703-545-155)
AlexaFluor 546 Donkey anti-Rabbit 1:1000 (Thermo Fisher Scientific, Cat# A10040)
AlexaFluor 647 Donkey anti-Rabbit ICC-IF 1:1000; IHC-IF 1:500 (Jackson Immuno Research Labs, Cat# 711-605-152)
AlexaFluor 647 Donkey anti-Mouse 1:1000 (Jackson Immuno Research Labs, Cat# 715-605-150)
Biotinylated donkey anti-rabbit 1:500 (Jackson Immuno Research Labs, Cat#711-065-152)
Biotinylated donkey anti-rat 1:200 (Jackson Immuno Research Labs, Cat#712-065-150)
Alexa Fluor 647 donkey anti-chicken 1:400 (Jackson Immuno Research Labs, Cat#703-605-155)
Cy™3-conjugated Fab Fragment Donkey Anti-Rabbit 1:2000 (Jackson Immuno Research Labs, Cat#711-167-003)
(FITC)-conjugated donkey anti-rabbit 1:500 (Jackson Immuno Research Labs, Cat#711-095-152)
(FITC)-conjugated donkey anti-goat 1:500 (Jackson Immuno Research Labs, Cat#705-095-147)
Cy™3-conjugated donkey anti-goat 1:400 (Jackson Immuno Research Labs, Cat#705-165-147)
Cy™3-conjugated donkey anti-guinea-pig 1:400 (Jackson Immuno Research Labs, Cat#706-165-148)
Cy™3-conjugated streptavidin 1:6000 (Jackson Immuno Research Labs, Cat#016-160-084)
(FITC)-conjugated streptavidin 1:1000 (Jackson Immuno Research Labs, Cat#016-010-084)
chicken anti-MAP2 1:1000 (Novus biologicals, Cat# NB300-213)

mouse mNeonGreen 1:500 (ChromoTek, Cat# 32F6)
mouse anti-SMI-312 1:1000 (HISS diagnostics, Cat# SMI-312R)
guinea pig VGluT1 1:500 (SySy, Cat# 135 304)
rabbit VGluT1 1:500 (SySy Cat# 135 308)
mouse anti-synaptophysin 1:100 (Leica Biosystems, Wetzlar, Germany, #PA0299)
mouse anti-neurofilament 200 kDa subunit 1:500 (NF-H) (Sigma, Saint Louis, MO, #N0142)
mouse anti-neurofilament 70 kDa subunit 1:500 (NF-L, Agilent, Santa Clara, CA, #M0762)
Mouse anti-SMI311 1:1000 (BIOZOL, Hamburg, cat#BLD-837801)
anti-GFP nanobody AF 488 1:500, (Nanotag, Göttingen, Cat# N0301)
Alpaca anti-VGluT1 nanobody 1:500 (Nanotag, Göttingen, cat# N1602-AF568-L)
goat anti-chicken Alexa 405 1:500 (Abcam Cambridge UK, cat# ab175674)
goat anti-rabbit STAR635P 1:1000 (Abberior, Göttingen Germany, ST635P Cat# 1002-500UG)
anti-mouse Alexa 750 1:1000 (ThermoFisher, Waltham USA, cat# A21037)
Alexa Fluor 568-conjugated polyclonal goat ant-rabbit 1:1000 (Life Techn. Carlsbad USA Cat #A-11036)
Alexa Fluor 750-conjugated polyclonal goat anti-guinea pig IgG H&L 1:500 (Abcam, #ab175758)
Alexa Fluor 568-conjugated polyclonal goat anti-mouse 1:1000 (Invitrogen #a-11004)

Validation

Chicken polyclonal anti-GFP, validated in previous refs, reported in manufacturer´s page https://www.antibodiesinc.com/products/anti-green-fluorescent-protein-antibody-gfp
Rabbit Polyclonal anti-RFP, validated in previous refs, reported in manufacturer´s page https://www.rockland.com/categories/primary-antibodies/rfp-antibody-pre-adsorbed-600-401-379/
Chicken polyclonal anti-MAP2, validated in previous refs, reported in manufacturer´s page https://www.abcam.com/en-us/products/primary-antibodies/map2-antibody-ab5392
Mouse polyclonal anti-vGlut1, validated in previous refs and in KO samples, reported in manufacturer´s page https://sysy.com/product/135311
Rabbit polyclonal anti-Homer1, validated in previous refs, reported in manufacturer´s page https://www.sysy.com/product/160003
Mouse polyclonal anti-Bassoon, validated in previous refs and in KO samples, reported in manufacturer´s page https://sysy.com/product/141111
CGRP antibody (Go Pc; Abcam, #ab36001) was previously validated on mouse lungs (10.1186/s12931-018-0915-8)
GAP43 antibody (Rb Pc; Novus Biologicals, #NB300-143) was validated by the manufacturer on mouse samples (https://www.novusbio.com/products/gap-43-antibody_nb300-143)
GFP antibody (Ch Pc; Abcam, #13970) was validated by the manufacturer on mouse samples (https://www.abcam.com/en-us/products/primary-antibodies/gfp-antibody-ab13970)
PGP9.5 antibody (Rb Pc; Abcam, #ab108986) was validated by the manufacturer on mouse samples (https://www.abcam.com/en-us/products/primary-antibodies/pgp95-antibody-epr4118-neuronal-marker-ab108986)
P2X3 antibody (Rb Pc; Chemicon, #AB5895) and SP antibody (Ra Mc; Biogenesis, 8450-0505) were previously validated on mouse lungs (10.1007/s00418-008-0495-7)
P2X3 antibody (Rb Pc; Chemicon, #AB5895) and SP antibody (Ra Mc; Biogenesis, 8450-0505) were previously validated on mouse lungs (10.1007/s00418-008-0495-7)
SYP antibody (GP Pc; Synaptic Systems, #101002) was validated by the manufacturer on mouse samples (https://sysy.com/product/101002)
VGLUT1 antibody (Rb Pc; Synaptic Systems, #135303) was validated by the manufacturer on mouse samples (https://sysy.com/product/135303)
Bassoon Mouse, 1:500, Enzo (New York, USA), ADI-VAM-PS003-F, cat# SAP7F407, validated 73 times, most recent: Yamamoto et. al., 2022 - Cell Biol.
AlexaFluor 488 anti-Chicken, validated in previous refs, reported in manufacturer´s page https://www.jacksonimmuno.com/catalog/products/703-545-155
Alexa 546 anti-Rabbit, validated in previous refs, reported in manufacturer´s page https://www.thermofisher.com/antibody/product/Donkey-anti-Rabbit-IgG-H-L-Highly-Cross-Adsorbed-Secondary-Antibody-Polyclonal/A10040
Alexa 647 anti-Rabbit, validated in previous refs, reported in manufacturer´s page https://www.jacksonimmuno.com/catalog/products/711-605-152
Alexa 647 anti-Mouse, validated in previous refs, reported in manufacturer´s page https://www.jacksonimmuno.com/catalog/products/715-605-150
Biotinylated donkey anti-rabbit IgG (1:500) validated in previous refs reported in manufacturer's page (https://www.jacksonimmuno.com/catalog/products/711-065-152)
Biotinylated donkey anti-rat IgG (1:200) validated in previous refs reported in manufacturer's page (https://www.jacksonimmuno.com/catalog/products/712-065-150)
Alexa Fluor® 647 donkey anti-chicken IgY (IgG) (1:400) validated in previous refs reported in manufacturer's page (https://www.jacksonimmuno.com/catalog/products/703-605-155)
Cy™3-conjugated Fab Fragment Donkey Anti-Rabbit IgG (1:2000) validated in previous refs reported in manufacturer's page (https://www.jacksonimmuno.com/catalog/products/711-167-003)
Fluorescein (FITC)-conjugated donkey anti-rabbit IgG (1:500) validated in previous refs reported in manufacturer's page (https://www.jacksonimmuno.com/catalog/products/711-095-152)
Fluorescein (FITC)-conjugated donkey anti-goat IgG (1:500) validated in previous refs reported in manufacturer's page (https://www.jacksonimmuno.com/catalog/products/705-095-147)
Cy™3-conjugated donkey anti-goat IgG (1:400) validated in previous refs reported in manufacturer's page (https://www.jacksonimmuno.com/catalog/products/705-165-147)
Cy™3-conjugated donkey anti-guinea-pig IgG (1:400) validated in previous refs reported in manufacturer's page (https://www.jacksonimmuno.com/catalog/products/706-165-148)
Cy™3-conjugated streptavidin (1:6000) validated in previous refs reported in manufacturer's page (https://www.jacksonimmuno.com/catalog/products/016-160-084)
Fluorescein (FITC)-conjugated streptavidin (1:1000) validated in previous refs reported in manufacturer's page (https://www.jacksonimmuno.com/catalog/products/016-010-084)
chicken anti-MAP2, validated in previous refs, reported in manufacturer's page https://www.novusbio.com/products/map2-antibody_nb300-213
mouse mNeonGreen validated in previous refs, reported in manufacturer's page https://www.ptglab.com/products/mNeonGreen-

antibody-32F6
SMI312 Mouse, 1:1000, HISS Diagnostics/Covance (Freiburg, Germany), cat# SMI-312R, validated in 100 citations, most recent: Abedin MJ, et al. 2023. Front Bioeng Biotechnol.
guinea pig VGluT1, SySy, Cat# 135 304, validated in manufacturer's page https://www.sysy.com/product/135304
rabbit VGluT1, SySy Cat# 135 308, KO validated, reported in manufacturer's page: https://www.sysy.com/product/135308
mouse anti-synaptophysin, Leica Biosystems, #PA0299 validated, reported in manufacturer's page https://shop.leicabiosystems.com/ihc-ish/ihc-primary-antibodies/pid-synaptophysin
mouse anti-neurofilament 200 kDa subunit NF-H, Sigma, #N0142, validated in 425 citations, most recent: Ke et al., 2025 CNS Neuroscience & Therapeutics
mouse anti-neurofilament 70 kDa subunit, NF-L Agilent, #M0762, validated in manufacturer's page https://www.labome.com/product/Dako/M0762.html
SMI311 Mouse, 1:1000, BIOZOL (Hamburg, Germany), cat# BLD-837801, validated in 17 citations, most recent: Yang J, et al. 2023. Brain Sci.
anti-GFP nanobody AF 488, Nanotag, Cat# N0301, validated in 23 citations, most recent: Shaib A et al, (2024) Nat. Biotec.
vGlut1 nbAZDye568 Alpaca, 1:500, Nanotag (Göttingen, Germany), cat# N1602-AF568-L, verified in 8 citations, most recent: Mougios et al., 2024 Nat. Com.
goat anti-chicken Alexa 405, Abcam Cambridge UK, cat# ab175674, validated, reported in manufacturer's page https://www.abcam.com/en-us/products/secondary-antibodies/goat-chicken-igy-h-l-alexa-fluor-405-ab175674
goat anti-rabbit STAR635P, Abberior, ST635P Cat# 1002-500UG validated in manufacturer's page https://abberior.shop/abberior-STAR-635P-goat-anti-rabbit-IgG-500-ll-1-mg-ml
anti-mouse Alexa 750, ThermoFisher, Waltham USA, cat# A21037) validated, reported in manufacturer's page: https://www.thermofisher.com/antibody/product/Goat-anti-Mouse-IgG-H-L-Cross-Adsorbed-Secondary-Antibody-Polyclonal/A-21037
Alexa Fluor 568-conjugated polyclonal goat ant-rabbit IgG H&L, cited in 2220 publications, with 55 published images. Applications used for ICC-IF, and IHC.
Alexa Fluor 750-conjugated polyclonal goat anti-guinea pig IgG H&L, from Abcam antibodies, cited in one publication (PMID: 33789950, DOI: 10.1681/ASN.2020101459). Applications include WB, ICC/IF, ELISA, IHC-P, Flow Cyt, IHC-Fr.
Alexa Fluor 568-conjugated polyclonal goat anti-mouse antibody, supplied by Invitrogen Antibodies, cited in 2603 publications, with 209 published images. Applications used include ICC-IF, IHC, IHC-IF, and IF.

Eukaryotic cell lines

Policy information about cell lines and Sex and Gender in Research

Cell line source(s)	Murine cell lines (AVR424.3 and RP1462) were isolated from murine tumors in the RP line, human cell lines (COR-L88, H1836, H69, H146, DMS273, H524, H211, H526, H1975, HCC44, HOP62, H2291, HEK293T) were gifts from professor Roman Thomas
Authentication	Cell lines were authenticated through genotyping (murine lines) and STR profiling (human lines).
Mycoplasma contamination	All cell lines tested negative for mycoplasma contamination
Commonly misidentified lines (See ICLAC register)	No commonly misidentified lines were used

Animals and other research organisms

Policy information about studies involving animals; ARRIVE guidelines recommended for reporting animal research, and Sex and Gender in Research

Laboratory animals	Animal experiments were performed with adult male and female mice derived from the following lines: Rb1-flox, Trp53-flox, Rosa26-LSL-PB, ATP1-S2, ATP1-H39, Thy1-GFP-M, Rosa26-Cas9-GFP, Rbl2-flox,Rosa26-LSL-tdTomato, H11-LSL-Cas9 and wild type C57BL/6. Wild type C57BL/6 mice were also used for neuronal preparation (adult mothers and embryos E13.5-16.5) and nodose ganglia preparations (3-5 weeks old mice).
Wild animals	This study did not involve wild animals
Reporting on sex	This study included animals of both sexes, but was not powered to detect sex-specific effects.
Field-collected samples	This study did not involve field-collected samples
Ethics oversight	The animals experiments performed in Cologne, Germany were approved by the Landesamt für Natur, Umwelt und Verbraucherschutz Nordrhein-Westfalen. For animal experiments performed at Stanford University, mice were maintained according to practices approved by the NIH, the Stanford Institutional Animal Care and Use Committee (IACUC), and the Association for Assessment and Accreditation of Laboratory Animal Care (AAALAC). The study protocol was approved by the Stanford Administrative Panel on Laboratory Animal Care (APLAC) (protocol 13565).

Note that full information on the approval of the study protocol must also be provided in the manuscript.

Plants

Seed stocks

Report on the source of all seed stocks or other plant material used. If applicable, state the seed stock centre and catalogue number. If plant specimens were collected from the field, describe the collection location, date and sampling procedures.

Novel plant genotypes

Describe the methods by which all novel plant genotypes were produced. This includes those generated by transgenic approaches, gene editing, chemical/radiation-based mutagenesis and hybridization. For transgenic lines, describe the transformation method, the number of independent lines analyzed and the generation upon which experiments were performed. For gene-edited lines, describe the editor used, the endogenous sequence targeted for editing, the targeting guide RNA sequence (if applicable) and how the editor was applied.

Authentication

Describe any authentication procedures for each seed stock used or novel genotype generated. Describe any experiments used to assess the effect of a mutation and, where applicable, how potential secondary effects (e.g. second site T-DNA insertions, mosiacism, off-target gene editing) were examined.
